# A Bednar tumor in a healthy woman

**DOI:** 10.1002/ccr3.3029

**Published:** 2020-06-15

**Authors:** Takayuki Yamada, Susumu Ohwada

**Affiliations:** ^1^ Asunaro Clinic Gunma Japan; ^2^ ASKOHWADA Consultation Clinic of Gastroenterology and Oncology Gunma Japan

**Keywords:** Dermatofibrosarcoma/diagnosis, Dermatofibrosarcoma/surgery, Fibrosarcoma/diagnosis, Immunohistochemistry, pigmented dermatofibrosarcoma protuberans, Skin neoplasm/surgery/diagnosis, slowly growing subcutaneous tumor

## Abstract

A Bednar tumor (BT) is a soft tissue sarcoma that mimics neurofibroma and rarely occurs in the deeper layers of the skin. Local recurrence of BT is possible with incomplete resection, but the occurrence of distant metastasis is rare.

## Graphical abstract

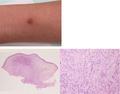

## 1 INTRODUCTION

A 48‐year‐old otherwise healthy woman presented with a 12‐month history of a slightly elevated nodular, nontender, foul‐smelling tumor, which bled when touched, on the left anterior forearm. The well‐demarcated, movable, brownish tumor was present since 12 months and had gradually increased in size to 15 mm.

What is your diagnosis?

A) Dermatofibroma, B) Neurofibroma, C) Nevus intradermal, D) Bednar tumor.

Answer: D

**Figure 1:**
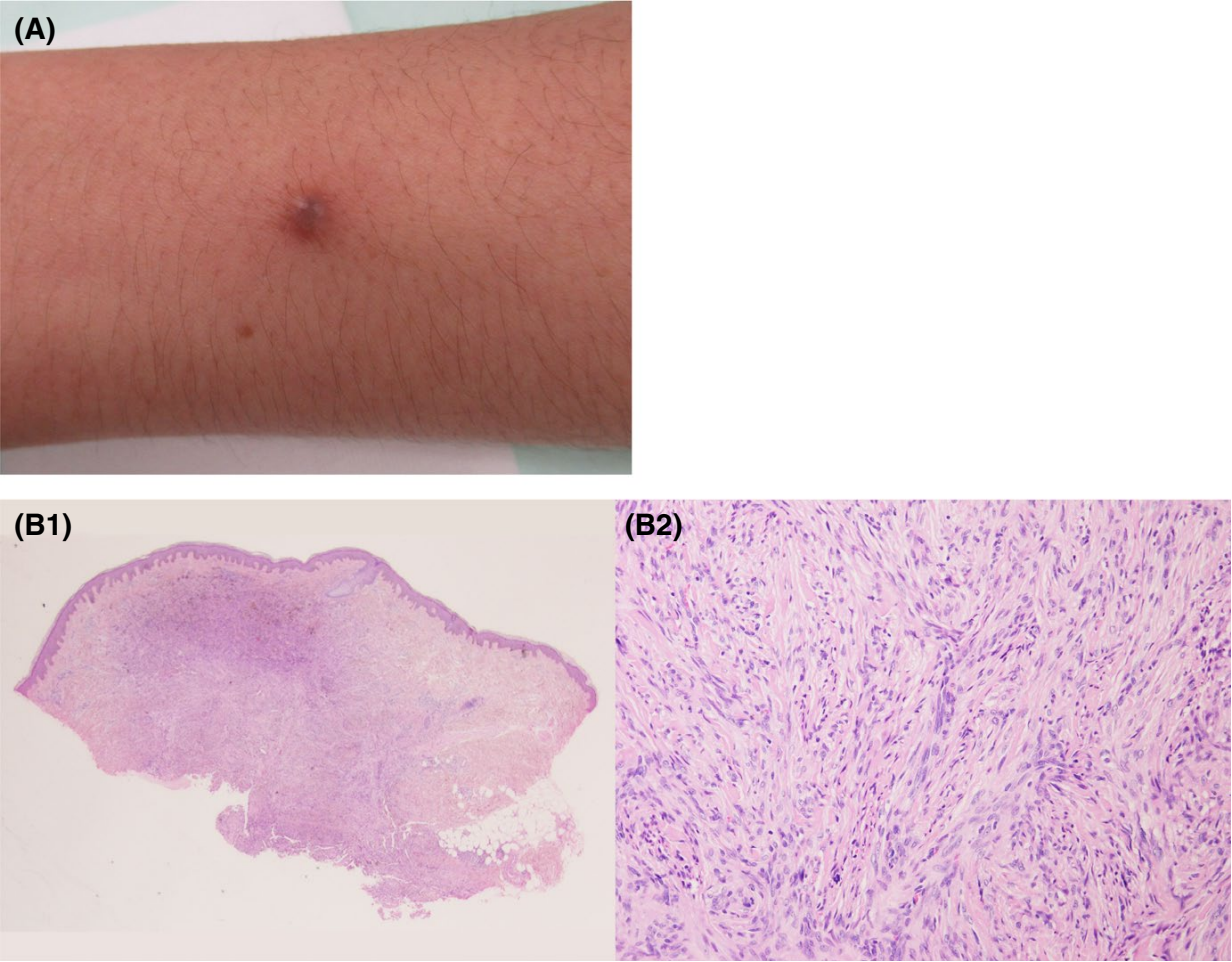
A, A slightly elevated nodular tumor existing on the left anterior forearm. B1, A circumscribed lesion with pigmentation seen at the periphery. B2, A storiform pattern of multinucleated spindle cell outgrowth admixed with few melanin‐containing dendritic cells

The clinical history and tumor shape suggested a neurofibroma (Figure 1A). A circumscribed lesion with pigmentation was seen at the periphery (Figure 1B1). A storiform pattern of multinucleated spindle cell outgrowth admixed with few melanin‐containing dendritic cells (Figure 1B2) confirmed pigmented dermatofibrosarcoma protuberans or Bednar tumor (BT).

A Bednar tumor is a soft tissue sarcoma that rarely occurs in the deeper layers of the skin and often presents as a small hard macula, violet or red‐colored, slow‐growing, and forms an elevated lesion that mimics neurofibroma.^1^ BT must be considered in case of any pigmented skin subcutaneous lesion, from benign ones including dermatofibroma to melanoma variants. Physicians should mention that the presence of pigment‐laden dendritic cells distinguishes this lesion from conventional dermatofibrosarcoma protuberans. Local recurrence of BT is possible with incomplete resection, but the occurrence of distant metastasis is rare.^1^

## CONFLICT OF INTEREST

None declared.

## AUTHOR CONTRIBUTIONS

TY served as a diagnostician and first author. SO served as a supervisory doctor.

## References

[1] Acosta AE , Vélez CS . Dermatofibrosarcoma protuberans. Curr Treat Options Oncol. 2017;18(9):56. 28795284 10.1007/s11864-017-0498-5

